# Оценка in vitro биологической активности отечественного препарата макрофаг-активирующего фактора (GcMAF-RF)

**DOI:** 10.18699/VJ20.621

**Published:** 2020-05

**Authors:** Е.В. Левитес, С.С. Кирикович, Е.В. Долгова, А.С. Проскурина, Г.С. Риттер, А.А. Останин, Е.Р. Черных, С.С. Богачев

**Affiliations:** Федеральный исследовательский центр Институт цитологии и генетики Сибирского отделения Российской академии наук, Новосибирск, Россия; Федеральный исследовательский центр Институт цитологии и генетики Сибирского отделения Российской академии наук, Новосибирск, Россия; Федеральный исследовательский центр Институт цитологии и генетики Сибирского отделения Российской академии наук, Новосибирск, Россия; Федеральный исследовательский центр Институт цитологии и генетики Сибирского отделения Российской академии наук, Новосибирск, Россия; Федеральный исследовательский центр Институт цитологии и генетики Сибирского отделения Российской академии наук, Новосибирск, Россия Новосибирский национальный исследовательский государственный университет, Новосибирск, Россия; Научно-исследовательский институт фундаментальной и клинической иммунологии, Новосибирск, Россия; Научно-исследовательский институт фундаментальной и клинической иммунологии, Новосибирск, Россия; Федеральный исследовательский центр Институт цитологии и генетики Сибирского отделения Российской академии наук, Новосибирск, Россия

**Keywords:** Gc protein-derived macrophage activating factor (GcMAF), vitamin D_3_-binding protein (DBP), phagocytosis, nitrogen monoxide (NO), peritoneal macrophages., макрофаг-активирующий фактор (GcMAF), витамин D_3_-связывающий белок (DBP), фагоцитоз, монооксид азота (NO), перитонеальные макрофаги.

## Abstract

В статье сообщается о разработанном оригинальном способе получения витамин D3-связывающего белка (DBP) и его конвертации в макрофаг-активирующий фактор GcMAF-RF. Согласно разработанному регламенту, DBP получали из плазмы крови человека, применяя аффинную колоночную хроматографию, очи- щали и модифицировали до GcMAF-RF с использованием цитоиммобилизованных гликозидаз (бета-галакто- зидаза и нейраминидаза). Принадлежность полученного полипептида к Gc-группе глобулинов плазмы крови подтверждали вестерн-блотом с использованием специфических антител. Полученный полипептид по своим молекулярным свойствам соответствует описанному в литературе белку GсMAF, находящемуся на стадии кли- нических испытаний в США, Британии, Израиле и Японии (Saisei Mirai, Reno Integrative Medical Center, Immuno Biotech Ltd, Efranat, Catalytic Longevity). Биологическую активность препарата GcMAF-RF определяли по индук- ции у перитонеальных макрофагов мыши фагоцитарной активности и способности продуцировать моноок- сид азота (NO) in vitro. Фагоцитарную активность макрофагов оценивали по эффективности захвата магнитных шариков. Степень активации макрофагов рассчитывали по отношению числа захваченных шариков к общему числу макрофагов. Уровень продукции NO оценивали по накоплению монооксида азота в культуральных су- пернатантах перитонеальных макрофагов колориметрическим методом с использованием реактива Грисса. Показано, что GcMAF-RF кратно увеличивает фагоцитарную активность макрофагов и достоверно увеличивает продукцию ими монооксида азота. Выделенный оригинальным способом активатор макрофагов GcMAF-RF по своим характеристикам (согласно материалам, опубликованным в печати) соответствует препаратам GcMAF, представляемым на рынке зарубежными компаниями, и может рассматриваться как новый отечественный био- логически активный препарат с широким спектром действия. Наибольший интерес вызывает его способность через активацию макрофагов усиливать адаптивный иммунитет организма. В этой связи предполагаются два направления терапевтического применения препарата GcMAF-RF. Препарат может быть востребован в области лечения онкологических заболеваний и, кроме того, может быть использован при лечении ряда нейродегене- ративных патологий и иммунодефицитных состояний.

## Abstract (en)

The article reports an original method for producing vitamin D3-binding protein (DBP) and its conversion into macrophage-activating factor GcMAF-RF. According to an original protocol, DBPs were obtained from human blood plasma using affinity chromatography, purified and modified to GcMAF-RF using cytoimmobilized glycosidases (beta-galactosidase and neuraminidase). The presence of the polypeptide obtained in the Gc group of blood plasma globulins was confirmed by Western blot using specific antibodies. The molecular properties of this polypeptide put it in correspondence with the GcMAF protein described in the literature, which is undergoing clinical trials in the USA, Britain, Israel and Japan (at Saisei Mirai; Reno Integrative Medical Center; Immuno Biotech Ltd; Efranat; and Catalytic Longevity). The biological activity of the GcMAF-RF preparation was detected by the induction of phagocytic activity of macrophages and their ability to produce nitrogen monoxide (NO) in vitro. The phagocytic activity of macrophages was evaluated by their ability to uptake magnetic beads. The degree of activation of macrophages was calculated by the ratio of trapped beads to the total number of macrophages. The level of NO production was estimated by the accumulation of nitrogen monoxide in the culture supernatants of peritoneal macrophages by the colorimetric method using the Griess reagent. It was shown that GcMAF-RF multiplies the phagocytic activity of macrophages and significantly increases their production of nitrogen monoxide. The macrophage activator GcMAF-RF, according to its characteristics, corresponds to similar preparations which are made available to the market by foreign companies, and can be considered as a new biologically active preparation with a wide spectrum of action. Of greatest interest is its ability – through the activation of macrophages – to enhance the adaptive immunity. In this regard, two areas of therapeutic use of the GcMAF-RF are proposed. The preparation will be in demand in the field of cancer treatment, and, in addition, it can be used in the treatment of a number of neurodegenerative pathologies.

## Введение

GcMAF (group-specific component protein-derived macrophage activating factor), важный компонент системы акти- вации макрофагов, образуется в результате сайт-специфического дегликозилирования витамин D3-связывающего белка (DBP), который присутствует в плазме крови человека в большом количестве (300–600 мг/л) (Malik et al., 2013; Delanghe et al., 2015). Нативный DBP содержит один трисахарид, ковалентно связанный с треонином в позиции 420 и состоящий из N-ацетилгалактозамина (GalNAc) с присоединенными к нему галактозой и сиало- вой кислотой. Преобразование DBP в GcMAF происходит путем отсоединения от GalNAc галактозы и сиаловой кислоты под действием β-галактозидазы и сиалидазы, ло- кализованных на клеточных мембранах активированных В- и Т-лимфоцитов соответственно (Yamamoto, Homma, 1991; Yamamoto, Kumashiro, 1993). В результате такого селективного дегликозилирования образуется активный белок GcMAF. Считается, что именно GalNAc, входящий в состав активного центра GcMAF, обеспечивает активацию макрофагов (Naraparaju, Yamamoto, 1994; Mohamad et al., 2002; Saburi et al., 2017a, b).

Исследовательский и практический интерес к GcMAF определяется его способностью через активацию макро- фагов участвовать в защитных реакциях организма: в за- щите от патогенов, в элиминации стареющих, опухолевых и поврежденных клеток, а также в процессах заживления. Широта биологических эффектов макрофагов, имеющих в ряде случаев оппозитную направленность, обеспечивается высокой функциональной гетерогенностью макрофагов (Gordon, 2003; Cassetta et al., 2011). Наиболее четко вы- деляются два субтипа макрофагов, которые обозначаются как М1- и М2-клетки с про- и противовоспалительной активностью соответственно.

М1-макрофаги играют важную роль в элиминации опу- холевых клеток. Они способны проявлять цитотоксиче- скую, микробицидную и антипролиферативную активно- сти, опосредованные продукцией активных метаболитов кислорода (например, Н_2_О_2_), монооксида азота (NO) и провоспалительных цитокинов. М2-макрофаги, напротив, проявляя противовоспалительную активность, ограничивают воспалительный/иммунный ответ. Повышенная активность М2-макрофагов сопряжена с развитием имму- носупрессии, приводящей к опухолевому росту (Lamagna et al., 2006; Sica, Bronte, 2007; Murray, Wynn, 2011).

Установлено, что функциональный тип макрофагов во многом определяется условиями их активации и инак- тивации (Korbelik et al., 1998; Mosser, 2003; Saburi et al., 2017a, b). Одним из факторов, препятствующих активации макрофагов, является ингибирование продукции GcMAF, осуществляемое ферментом нагалазой (α-N-ацетилгалактозаминидазой), секретируемым опухолевыми клетками (Korbelik et al., 1998; Rehder et al., 2009; Saburi et al., 2017a, b). Сывороточная нагалаза у больных раком способна полностью дегликозилировать предшественник MAF (DBP), осуществляя гидролиз по GalNAc-остатку. Лишенный активного сайта, полипептид теряет способность активировать инфильтрующие опухоль макрофаги, что в клинических наблюдениях характеризуется как иммуносупрессия, связанная с потерей макрофагами специфических активностей (Yamamoto et al., 1996; Mohamad et al., 2002; Matsuura et al., 2004; Thyer et al., 2013a). У здоровых людей уровень нагалазы в несколько раз ниже, чем у онкологических больных, и нагалаза в отсутствие патологии не дегликозилирует трисахарид DBP (Ioannou et al., 1992; Nagasawa et al., 2005).

В пионерных работах N. Yamamoto (Yamamoto, Homma, 1991; Yamamoto, Kumashiro, 1993; Yamamoto et al., 1996) было сделано предположение, что инъекции очищенного экзогенного GcMAF могут компенсировать дефектный фактор, активировать систему макрофагов и их противораковую активность. Проведенные клинические исследования свидетельствовали об эффективном воздействии GcMAF на опухоль, приводящем к значительной редукции опухолевого очага или полному уходу опухоли с продолжительным (несколько лет) безрецидивным периодом (Yamamoto et al., 2008; Rehder et al., 2009; Inui et al., 2013; Thyer et al., 2013a, b).

Со времени опубликования N. Yamamoto своих результатов относительно принадлежности выделенного полипептида к групп-специфическому активатору макрофагов прошло более 15 лет. Исследованием полипептида в направлении поиска мишеней его клинического применения занимались разные лаборатории. Получены многочис- ленные, противоречивые данные о его функциональных возможностях в качестве активатора иммунных реакций при лечении злокачественных новообразований, аутизма, различных нарушений в работе иммунной системы. Противоречивые результаты, касающиеся эффективности клинических возможностей GcMAF, вызвали немалую долю скептицизма в научном сообществе (Rehder et al., 2009; Ugarte et al., 2014; Borges, Rehder, 2016; Ruggiero et al., 2016). Такое состояние вопроса связано еще и с тем, что препарат невозможно сделать предметом промышленной собственности, а можно только патентовать различные способы его получения и различные композиции, в составе которых он может применяется. Именно по такому пути идут все производители GcMAF (Saisei Mirai, Reno Integrative Medical Center, Immuno Biotech Ltd, Efranat, Catalytic Longevity). Тем не менее о перспективности возможного практического использования препарата GcMAF свидетельствуют многочисленные данные, полученные на экспериментальных животных, а также данные доклинических исследований и накопленный положительный опыт его клинического применения (Korbelik et al., 1997; Kisker et al., 2003; Yamamoto et al., 2008; Toyohara et al., 2011; Pacini et al., 2012; Inui et al., 2013, 2016a, b; Kuchiike et al., 2013; Thyer et al., 2013a, b; Klokol, Teppone, 2016; Saburi et al., 2017a, b; Moya et al., 2018; Păduraru et al., 2019; Greilberger, Herwig, 2020).

Проведенный нами анализ литературных источников продемонстрировал большой интерес к препарату GcMAF в мире, несмотря на имеющиеся различные точки зрения (Останин и др., 2019), и определил направление его исследования в нашей лаборатории. Поскольку практически во всех исследованиях процедура получения и активации препарата переписывается с одной-двух пионерных работ (Link et al., 1986; Yamamoto, Homma, 1991; Yamamoto, Kumashiro, 1993), что связано с его коммерческой перспективностью и нежеланием раскрывать детали выделения больших количеств активатора, мы решили разработать независимый регламент получения и активации фактора и экспериментально определить его возможную «клиническую мишень».

В настоящей работе, представляющей собой первую статью цикла из трех статей, описывающих действие препарата на экспериментальные биологические системы, оценивается способность полученного оригинальным способом препарата GcMAF-RF (GcMAF-Related Factor) активировать фагоцитарную функцию макрофагов и продуцировать монооксид азота in vitro.

Оригинальный способ выделения DBP и процедура его конвертации в GcMAF-RF цитоэнзиматическим способом были разработаны в ООО «Активатор MAF» совместно с лабораторией индуцированных клеточных процессов Федерального исследовательского центра Институт цитологии и генетики (ФИЦ ИЦиГ) СО РАН. Способ получения препарата GcMAF-RF, в связи с его статусом «предмета промышленной собственности» компании ООО «Активатор MAF», охарактеризован здесь без указания деталей процедур. Тем не менее общая характеристика способа, представленная в разделе «Результаты», дает достаточно полную информацию, демонстрирующую оригинальность подхода. По своим молекулярным свойствам полученный полипептид соответствует описанному в литературе белку GсMAF, находящемуся на стадии клинических испытаний в США, Британии, Израиле и Японии (Saisei Mirai, Reno Integrative Medical Center, Immuno Biotech Ltd, Efranat, Catalytic Longevity).

## Материалы и методы

В экспериментах использовали по две-три месячных мыши линии СВА разведения вивария № 2 ФИЦ ИЦиГ СО РАН (стандартное содержание). Перитонеальные макрофаги (5 × 10^5^ клеток/лунку) культивировали в 12-луночных планшетах в среде RPMI-1640 (Biolot), содержащей 10 % FBS (HyClone) и 40 мкг/мл гентамицина в течение 12 ч. Затем адгезивную фракцию макрофагов три раза отмывали забуференным физиологическим раствором (PBS) для удаления неприкрепленных клеток. Полученные макрофаги использовали в дальнейшем для анализа их биологической активности.

Фагоцитарную функцию макрофагов оценивали согласно методике, представленной в работе (Ishikawa et al., 2014). Перитонеальные макрофаги выделяли из брюшной полости двух-трех мышей, объединяли, распределяли по лункам планшета в равном количестве и культивировали в бессывороточной среде RPMI-1640 в течение 2 ч. Затем среду меняли на RPMI-1640, содержащую 10 % FBS в отсутствие (контроль) или в присутствии следующих активаторов (позитивный контроль): липополисахарида (LPS, Sigma, 10 мкг/мл, Е. coli 0114:B4) либо полученных нами DBP (5 мкг/мл) или GcMAF-RF (5 мкг/мл). В каждую лунку добавляли также магнитные шарики (Dynabeads M-280, Invitrogen) в дозе 60 мкг/лунку. После трехчасовой инкубации макрофаги три раза отмывали PBS для удаления неинтернализованных шариков, затем фотографировали в проходящем свете с использованием инвертированного микроскопа AxioObserver Z1 (Zeiss) и подсчитывали количество интернализованных гранул (IBN). Фагоцитарную активность макрофагов оценивали по формуле: IBN = количество интернализованных шариков/ количество макрофагов. Для статистического анализа IBN учитывали данные четырех независимых экспериментов, в каждом эксперименте оценивали 300–500 клеток. Учет клеток был проведен из нескольких полей, расположенных в разных частях лунки планшета.

Продукцию NO определяли в пяти повторностях на семи мышах по накоплению нитритов после 3 ч инкубирования с активаторами в культуральных супернатантах перитонеальных макрофагов колориметрическим методом с использованием реактива Грисса (Green et al., 1982). Для этого 100 мкл каждого тестируемого супернатанта переносили в 96-луночный планшет, смешивали с равным объемом реактива Грисса и инкубировали при комнатной температуре в течение 15 мин. Оптическую плотность оценивали на многоканальном спектрофотометре при длине волны 540 нм. Результаты соотносили со стандартной калибровочной кривой, полученной на основе серийных разведений 3 мМ раствора нитрита натрия.

Статистический анализ проводили с использованием программного обеспечения Statistica 10. В каждом эксперименте было выполнено минимум четыре повторения. Существование статистически значимых различий между исследуемыми группами проанализировано при помощи критерия Краскела–Уоллиса. Для апостериорных сравнений между группами использовали U-критерий Манна–Уитни с учетом поправки Бонферрони (минимальный уровень значимости p = 0.05/ число сравнений). Таким образом, в случае анализа IBN различия считали достоверными при уровне значимости p < 0.017 (три попарных сравнения), а в случае анализа продукции NO – при p < 0.013 (четыре попарных сравнения) (Гржибовский, 2008).

## Результаты

Как сказано выше, витамин D_3_-связывающий белок содержит три функциональных сайта: витамин D_3_-связывающий домен, актин-связывающий сегмент полипептидной цепи и сайт гликозилирования. Соответственно, существуют два очевидных способа аффинного выделения специфического белка, за актин- и витамин D3-связывающие домены (Haddad et al., 1984; Link et al., 1986; Swamy, Ray, 1995). В исследованиях других авторов описывается практически всегда один и тот же способ получения активатора макрофагов GcMAF. Витамин D3 модифицируется в производную молекулу, содержащую гидроксил в положении 25, или химическим, или энзиматическим способом. Модифицированный витамин «пришивается » к активированной бромцианом сефарозе, и проводится аффинная хроматография. Далее белок активируется в GcMAF энзиматической конвертацией двумя гидролазами – салидазой и β-галактозидазой, ковалентно фиксированными на носителе (Yamamoto, Kumashiro, 1993; Yamamoto, 1996; Mohamad et al., 2002). Функциональная активность полученного GcMAF тестируется по его способности индуцировать у макрофагов способность фагоцитировать разнообразные внеклеточные частицы. Главным образом используются опсонированные эритроциты барана (Hammarstrom, Kabat, 1971; Yamamoto, Kumashiro, 1993). Наши многочисленные попытки выделить, активировать и оценить функциональную активность полученного полипептида способами, описанными в статьях, не увенчались успехом. Основными причинами неудач были: невозможность в достаточном количестве быстро получить составляющие компоненты всех сложных процедур, их высокая цена, постоянное утаивание авторами опубликованных работ принципиальных технических деталей той или иной процедуры.

В этой связи после тотальной проработки принципов получения GcMAF мы разработали следующий регламент выделения большого количества препарата витамин D_3_-связывающего белка, его конвертации в GcMAF-RF и оценки его способности активировать фагоцитарную функцию макрофагов и продукцию ими NO

Витамин D_3_-связывающий белок был выделен из сыворотки крови здоровых доноров с использованием аффинной хроматографии на колонке с ковалентно пришитым актином. Данный подход состоял из двух процедур. Во первых, актин, необходимый в качестве аффинного лиганда, получали самостоятельно из мышц кролика (Spudich, Watt, 1971), благодаря чему удалось быстро наладить аффинную хроматографию и на порядки сократить затраты. Одновременно из крабовых панцирей был выделен субстрат, необходимый для пришивки аффинного лиганда (de Souza et al., 2008). Этот способ также позволил не опираться на импортные дорогие реактивы и существенно ускорил время получения фактора. Для конвертации DBP в GcMAF-RF был разработан оригинальный способ с использованием активированных лизофосфатидил- холином (Lyso-Pc, Sigma) воспалительных лимфоцитов, получаемых от того же донора (Ngwenya, Yamamoto, 1986; Yamamoto, Homma, 1991; Asaoka et al., 1992). Суть подхода состоит в том, что на цитоплазматической мембране активированных к воспалению В- и Т-лимфоцитов присутствуют оба необходимых для конвертации DBP в GcMAF фермента: β-галактозидаза и салидаза соответственно. Полученные от донора лимфоциты после воспалительной активации добавлялись к DBP. После инкубации полученный GcMAF-RF проверялся на способность активировать специфическую фагоцитарную активность макрофагов. Для этого был валидирован способ с использованием металлических бус (Ishikawa et al., 2014). Процедура хорошо стандартизируется, не требует многостадийного получения опсонированных эритроцитов барана и высокотехнологична. Дополнительно для оценки специфичности выделенного GcMAF-RF был разработан оригинальный метод получения лектина, позволяющий оценить эффективность отщепления хвостов сахаров и сохранения в сайте гликозилирования N-ацетилгалакозамина, который является основной молекулой, участвующей в активации макрофагов.

Для проверки соответствия DBP, выделенного с использованием в качестве аффинного лиганда актина, препарату DBP, выделяемому аффинно на 25-гидроксивитамин D3-сефарозе, и характеристики принадлеж- ности обоих полипептидов к группе специфических Gc-белков был про- веден сравнительный вестерн-блот анализ. Прямой сравнительный вестерн- блот анализ образцов препарата с антителами против Gc-группы свидетельствует об идентичности упомя- нутых двух вариантов белков (рис. 1). 

**Fig. 1.**
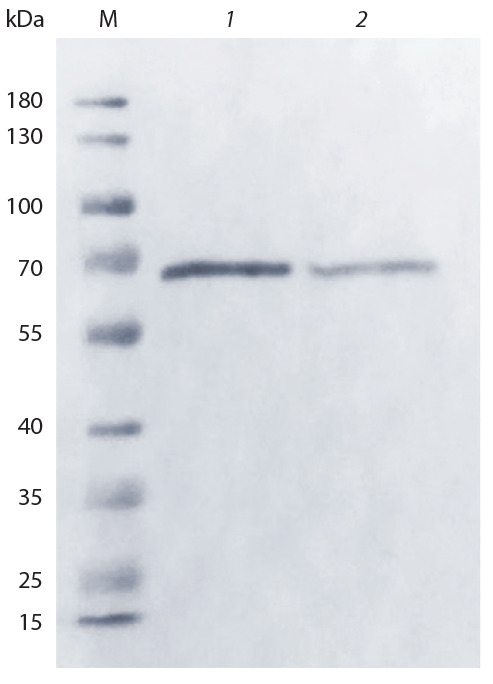
Western blot analysis of DBP samples obtained by actin and sepharose (with 25-hydroxyvitamin D_3_) chromatography. Lanes: M, molecular marker “The Thermo Scientific ™ Page Ruler™ Prestained protein Ladder” (Thermo Fisher Scientific Inc., USA); 1, DBP obtained on a column with 25-hydroxyvitamin D3-Sepharose; 2, DBP obtained by actin-sepharose affinity chromatography.

После получения, конвертации и проверки специфической активности, способности активировать фагоцитарную активность макрофагов, препарат GcMAF-RF стерилизовали фильтрованием, а затем или замораживали и хранили при –70 °С, или лиофилизировали (см. рис. 1).

Биологическую активность GcMAF-RF оценивали по его влиянию на фагоцитарную функцию перитонеальных макрофагов (Ishikawa et al., 2014) (рис. 2). Было выполнено три апостериорных сравнения экспериментальных групп с контролем с использованием U-критерия Манна–Уитни с учетом поправки Бонферрони. Показано, что по сравнению с контролем только препарат GcMAF-RF статистически значимо усиливает способность макрофагов интернализировать магнитные шарики. В присутствии GcMAF-RF фагоцитарная активность макрофагов увеличивалась в 3.7 раза ( p = 0.011), тогда как в ответ на DBP или ЛПС – в 1.2 и 1.6 раза соответственно. Репрезентативные фотографии макрофагов (см. рис. 2, б ) четко демонстрируют, что при стимуляции препаратом GcMAF-RF (но не его предшественником DBP) в общей популяции перитонеальных макрофагов значительно возрастает число клеток с интернализованными магнитными шариками. В данном тесте оценивали биологическую активность образцов из каждой полученной партии препарата, при этом различные образцы GcMAF-RF демонстрировали трех-семикратное увеличение фагоцитарной функции макрофагов.

**Fig. 2.**
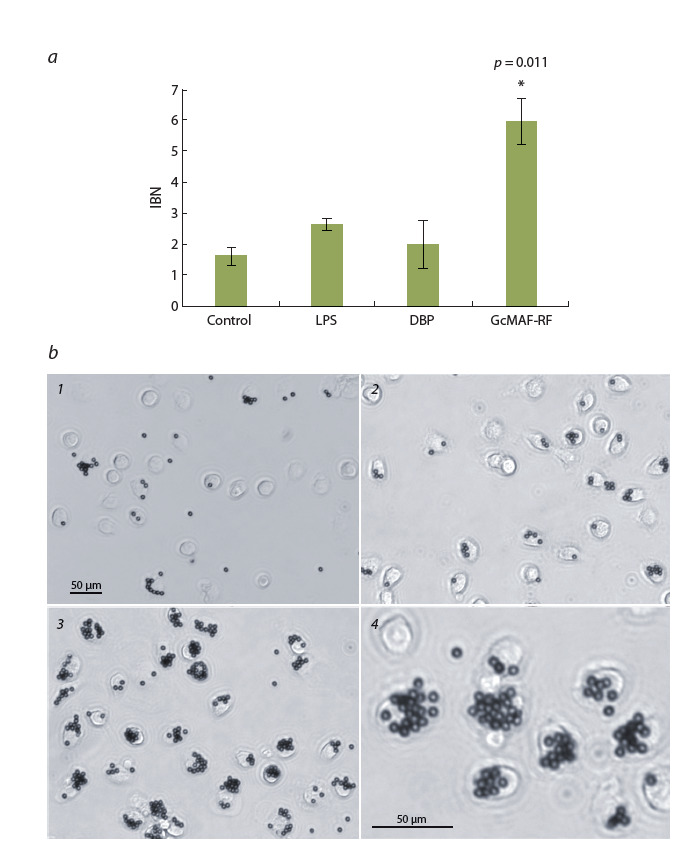
Effect of GcMAF-RF on the phagocytic activity of peritoneal macrophages. а, data are presented as М ± SEM (n = 4), * p < 0.017 – significance of differences compared with control (Bonferroni-corrected Mann–Whitney U-test); b, representative photographs of macrophages with internalized granules: 1, control; 2, after activation by DBP; 3, 4, after activation by GcMAF-RF.

Препарат GcMAF-RF усиливал не только фагоцитарную активность перитонеальных макрофагов, но также их способность продуцировать NO (рис. 3). Было проведено четыре апостериорных сравнения (экспериментальные группы с контролем и ЛПС и GcMAF-RF между собой) с использованием U-критерия Манна–Уитни с учетом поправки Бонферрони. Оказалось, что GcMAF-RF (но не его предшественник DBP) статистически значимо ( p < 0.013) повышал уро- вень продукции монооксида азота, при этом уровень индуцированной NO- продукции был даже выше, чем в ответ на стандартный активатор макрофагов ЛПС ( p = 0.008).

Все перечисленные процедуры, ва- лидирующие препарат как активатор макрофагов (GcMAF-RF), постоянно проводятся для характеристики каж- дого нового выделения препарата и доведения до состояния технологии, которая готовится к сертификации.

## Обсуждение

В настоящем исследовании проведена оценка биологической активности первого отечественного препарата GcMAF-RF, который был вы- делен из плазмы крови человека в соответствии с новым технологическим регламентом, разработанным компанией OOO «Активатор MAF». Прямой сравнительный вестерн-блот анализ образцов препарата с антителами против Gc-группы показал (см. рис. 1), что молекулярная масса белков, выделенных двумя вариантами аффинной хроматографии, соответствует размеру GcMAF в 65–67 кДа, определенному другими авторами в аналогичной трис-глициновой электрофоретической системе (Smith et al., 2013). Полученный результат свидетельствует об идентичности полипептидов, выделяемых с использовани-ем двух имеющихся в молекуле DBP доменов, различающихся по своей функциональной специфичности (актин- связывающий и витамин D_3_-связывающий).

В витральных тестах образцы GcMAF-RF из каждой полученной партии препарата проявляли свойства, харак- терные для макрофаг-активирующего фактора, а именно: кратно усиливали фагоцитарную функцию перитонеаль- ных макрофагов мыши (см. рис. 2), а также статистически значимо стимулировали продукцию NO (см. рис. 3).

**Fig. 3.**
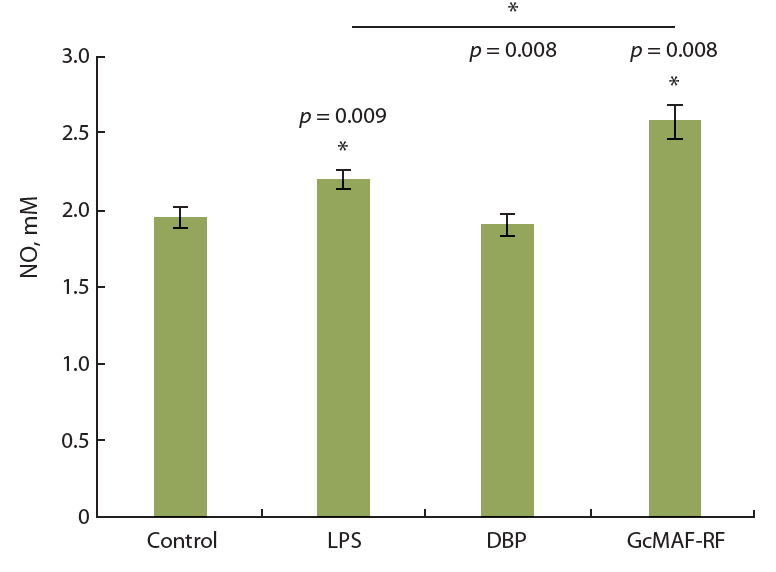
Effect of the drug GcMAF-RF on the production of NO. Data are presented as М ± SEM (n = 4). * Differences from the control and between the LPS and GcMAF-RF groups are significant at p < 0.013 (Bonferroni- corrected Mann–Whitney U-test).

Выявленные в нашем исследовании факты стимули- рующего влияния полученного препарата GcMAF-RF на свойства макрофагов согласуются с данными целого ряда исследователей (Mohamad et al., 2002; Thyer et al., 2013b; Ishikawa et al., 2014; Ruggiero et al., 2014; Saburi et al., 2017a, b). Это позволяет заключить, что препарат GcMAFRF соответствует известным импортным аналогам не только по своим физико-химическим характеристикам, но и по проявляемой биологической активности.

Следующие два сообщения будут содержать результаты по влиянию препарата GcMAF-RF на культуру дендрит- ных клеток и поляризацию М2-макрофагов. Также будет продемонстрирована способность активированных пре- паратом GcMAF-RF перитонеальных макрофагов лизи- ровать клетки нескольких опухолевых культур и оценена его противораковая активность в биологических тестах на экспериментальных животных.

## Заключение

Представлены первые экспериментальные данные, характеризующие способность препарата GcMAF-RF, полученного оригинальным способом, активировать перитонеальные макрофаги мыши, что проявляется кратным усилением их фагоцитарной функции и значимым по-вышением уровня продукции монооксида азота. Выделенный активатор макрофагов (GcMAF-RF) по своим характеристикам соответствует аналогичным препаратам, представляемым на рынке зарубежными компаниями, и может рассматриваться как новый отечественный биологически активный фактор разнонаправленного действия.

## Conflict of interest

The co-authors of the paper A.S. Proskurina and S.S. Bogachev are directors of Activator MAF and BA Pharma, respectively.
